# Seven-year outcomes of combined treatment of anti-vascular endothelial growth factor with photodynamic therapy for polypoidal choroidal vasculopathy; according to polypoidal lesion regression

**DOI:** 10.1186/s12886-023-03264-x

**Published:** 2023-12-14

**Authors:** Junwoo Lee, Kiyoung Kim, Eung Suk Kim, Seung-Young Yu, Min Seok Kang

**Affiliations:** 1 Department of Ophthalmology, Kyung Hee University Hospital, 23, Kyungheedae-ro, Dongdaemun- gu, 02447 Seoul, Republic of Korea; 2 Department of Medicine, Graduate School, Kyung Hee University, Seoul, Korea

**Keywords:** Polypoidal choroidal vasculopathy, Polyp regression, Anti-VEGF, Photodynamic therapy

## Abstract

**Purpose:**

To evaluate the long-term prognosis of polypoidal choroidal vasculopathy (PCV) treated with anti-vascular endothelial growth factor (anti-VEGF) combined with verteporfin photodynamic therapy (PDT), according to polypoidal lesion regression.

**Methods:**

This study retrospectively reviewed the data of 33 naïve eyes with PCV treated with anti-VEGF combined with verteporfin PDT and followed-up for at least 7 years. The collected data included demographic profile, best-corrected visual acuity (BCVA), central foveal thickness (CFT), PED volume, and presence of submacular hemorrhage. Regression of polypoidal lesion was determined using indocyanine green angiography and optical coherence tomography. All eyes were divided into regression or persistent groups, based on the polypoidal lesion regression one year after the initial combined treatment.

**Results:**

BCVA improvement was maintained for 3 years in the regression (*p* = 0.001) and 1 year in the persistent (*p* = 0.006) groups, respectively. The mean BCVA of the regression group was better than that of the persistent group over 7 years, but the difference was significant only at 1 year (*p* = 0.037). The number of eyes which maintained BCVA less than or equal to 0.3 logMAR at 7 years was 11 eyes (64.7%) in regression group and 4 eyes (25.0%) in persistent group (*p* = 0.022).

**Conclusions:**

Regression of the polypoidal lesion at 1 year after the initial combination treatment was associated with favorable long-term visual prognosis, particularly in terms of maintaining good visual acuity.

## Introduction

Polypoidal choroidal vasculopathy (PCV) is characterized by an orange-red protrusion on fundus images and polyp-like pooling of dye on indocyanine green angiography (ICGA) [1, 2]. The prevalence is higher in Asians compared to Caucasians, with up to 50% of cases occurring in Asian patients with age-related macular degeneration (AMD) [3, 4]. In Korea, PCV has been identified in 24.6% of patients with AMD [5].

PCV generally has a good prognosis compared to choroidal neovascularization (CNV). However, chronic progression of PCV may be observed, leading to changes in the retinal pigment epithelium, hemorrhage, or disciform scar formation, which can result in permanent visual loss [2, 4, 6, 7]. Thus, effective treatment is crucial, particularly for Asian populations with a high prevalence of PCV.

The key to PCV treatment is the occlusion of active polyps and resolution of exudative lesions. Photodynamic therapy (PDT) is a highly effective for occluding polypoidal lesions [8]. A recent study on the 5-year efficacy of PDT for PCV demonstrated significant visual improvement compared to the baseline, which was maintained up to 4 years after the treatment. The final outcome showed a stabilized or improved visual outcome in 88.1% of patients [9].

Regarding the combination treatment of anti-vascular endothelial growth factor (anti-VEGF) and verteporfin PDT, a significantly higher effect on polyp regression was found with intravitreal ranibizumab combined with verteporfin PDT or verteporfin PDT monotherapy than with ranibizumab monotherapy in an EVEREST study that compared the treatment effects for 6 months. The outcome of polyp regression was far better with combination treatment than with verteporfin PDT monotherapy [10]. Lai et al. reported stabilization of visual acuity in six out of seven eyes (85.7%) after combination treatment for PCV [11]. A recent study assessing the 36-month clinical outcomes associated with the combination treatment of PDT and anti-VEGF in patients with PCV reported that a decrease in visual acuity could be prevented in 88.2% of the patients [12]. Mio et al. reported that polypoidal regression and recurrence, incomplete polyp regression, and no regression on ICGA after treatment were associated with a higher recurrence rate of PCV [13]. Therefore, combination therapy of anti VEGF and verteporfin PDT is effective treatment for maintaining good visual prognosis and reducing the recurrence rate of PCV.

Although polyp regression is an important anatomical factor for the treatment of PCV, previous studies have shown an inconsistent correlation between polyp regression and visual outcomes, and there is a paucity of research on the long-term clinical course. [10, 14–16] In this study, we aimed to investigate the long-term visual outcome in PCV patients who received PDT combined with intravitreal anti-VEGF injection as the initial treatment, according to polyp regression.

## Methods

### Study design and data analysis

This retrospective study investigated 33 eyes of 31 patients who received a combination treatment of verteporfin PDT and intravitreal anti-VEGF injection following the diagnosis of PCV at Kyung Hee University Hospital. The patients were followed up for at least 7 years. Participant selection was based on a chart review.

The inclusion criteria were as follows: (1) symptomatic macular PCV, (2) presence of exudative or hemorrhagic features involving the macular on fundus examination, and (3) presence of polypoidal lesions and branching vascular networks (BVNs) on indocyanine green angiography (ICGA). The exclusion criteria were as follows: (1) presence of any systemic contraindications for verteporfin or angiographic dyes, (2) previous treatment for PCV, (3) presence of other diseases such as age-related macular degeneration or pathologic myopia, (4) retinal pigment epithelial tears, and (5) other maculopathies such as diabetic maculopathy or retinal vascular occlusion.

PCV was diagnosed based on the presence of polypoidal lesions and BVNs observed in the ICGA image. Images were obtained using the Heidelberg Retina Angiography System (Heidelberg Engineering, Heidelberg, Germany), equipped with a confocal scanning laser ophthalmoscope. Polyp regression was evaluated using ICGA and optical coherence tomography (OCT) 1-year after the initial treatment. Polyp regression was defined as the disappearance of polypoidal lesions observed on ICGA, accompanied by a decrease in the lesion size detected on OCT. According to polyp regression, eyes were classified into regression, and persistent group by two retinal specialists (ESK, KK). Two spectral-domain OCT devices (Spectralis, Heidelberg Engineering, Heidelberg, Germany, and Cirrus HD-OCT, Carl Zeiss Meditec, Dublin, California, USA) were used for evaluating polypoidal lesion during follow-up period. To measure the central foveal thickness (CFT), a vertical line was drawn using built-in caliper of Heidelberg Eye Explorer software to determine the distance between the Bruch’s membrane and the internal limiting membrane layer. For the evaluation of pigment epithelial detachment (PED) volume, the retinal pigment epithelium (RPE) elevation map algorithm of Cirrus HD-OCT was used.

At each follow-up, a comprehensive examination, including slit lamp examination, color fundus photography (CFP), OCT, and if necessary, ICGA was performed. ICGA was performed again after 1-year visit to assess polyp activity if reactivation of polyp was suspected based on fundus examination and OCT. In the first year after treatment, we conducted monthly follow-up and subsequently extended the intervals to every three months. For patients in a stable state, the follow-up interval was further extended to a maximum of 6 months.

Visual acuity was converted to the logarithm of the minimum angle of resolution (logMAR) scale for statistical analysis. Slit lamp examination and fundus examination were performed at the initial visit and subsequently at 3, 6, 12, 24, 36, 48, 60, 72, and 84 months of follow-up. To determine good visual acuity, the thresholds were set at 20/40, which is equivalent to 0.3 logMAR, as suggested by the World Health Organization (WHO) and the Centers for Disease Control and Prevention (CDC) as indicative of good vision [17].

This study was conducted in accordance with the principles of the Declaration of Helsinki. The protocol was approved by Kyung Hee University Hospital Institutional Review Board (2021-08-027), and the need for informed consent was waived due to the minimal risk of disclosing personal information.

### Treatment

PDT was performed based on ICGA image. All patients received an injection of 6 mg/m^2^ verteporfin (Visudyne; Novartis AG, Bulach, Switzerland) for 10 min, and after 15 min infusion, the area of the polyp was irradiated using a 689 nm diode laser. The lesion was irradiated with 50 J/cm^2^ light energy at a dose rate of 600 mW/cm^2^ 83 s. The diameter of the laser spot was calculated by adding the greatest linear dimension (GLD) plus 1 mm for all eyes.

The first intravitreal anti-VEGF injections were administered within seven days of PDT, followed by two consecutive injections. For the intravitreal injection, either bevacizumab (Avastin; Genentech Inc., San Francisco, CA) or ranibizumab (Lucentis; Genentech Inc.) was used. The injection was performed into the vitreous cavity using a 30-gauge needle, inserted form an area 3.5 mm posterior to the corneal limbus, following topical anesthesia. Informed consent was obtained from the patients for the off-label use of bevacizumab, with detailed explanation provided before the injection.Retreatment was determined based on ICGA, and OCT results. In cases which ICGA showed a polypoidal lesion accompanied by exudative changes, combination treatment of PDT and intravitreal anti-VEGF injection was administered. For cases with exudative changes alone, without a clearly defined polypoidal lesion, only intravitreal anti-VEGF injection was administered.

### Statistical analysis

Data analyses were performed using SPSS version 23.0 (SPSS Inc., Chicago, Ill., USA). The BCVA and CFT were compared with post-treatment values using the Wilcoxon signed-rank test. The regression and persistent groups were compared using the Mann-Whitney U test. The visual acuity of the two groups was compared using the Fisher’s exact test. Statistical significance was defined as *p* < 0.05.

## Results

### Baseline characteristics

Thirty-three eyes of 31 patients were examined for 7-year outcomes based on polyp regression. The average age of the patients was 66.0 ± 10.9 years. One year after the first treatment, polyp regressed in 17 eyes (regression group), and persisted in 16 eyes (persistent group). There were no significant differences between regression and persistent groups in age, BCVA, CFT, and PED volume at the baseline (Table 1).

**Table 1:** Baseline characteristics of all eyes, regression, and persistent groups

	Total	Regression	Persistent	*p*-value
No. of eyes	33	17 (51.5%)	16 (48.5%)	
Women	9 (27.3%)	3 (17.6%)	6 (37.5%)	0.259
Age (years)	66.0 ± 10.9	65.6 ± 9.1	66.4 ± 12.8	1.000
Diabetes	7 (21.2%)	4 (23.5%)	3 (18.8%)	1.000
Hypertension	18 (54.5%)	8 (47.1%)	10 (62.5%)	0.491
BCVA (logMAR)	0.58 ± 0.36	0.56 ± 0.57	0.59 ± 0.41	0.958
CFT (µm)	342.6 ± 199.1	345.6 ± 124.8	339.6 ± 117.1	0.897
PED vol (mm^3^)	0.36 ± 0.45	0.35 ± 0.54	0.38 ± 0.34	0.305
GLD (µm)	3737 ± 1095	3676 ± 1206	3801 ± 998	0.709

BCVA = best corrected visual acuity, CFT = central foveal thickness, PED = pigment epithelial detachment, GLD = greatest linear dimension, SMH = submacular hemorrhage

### Visual outcomes

**Fig. 1:**
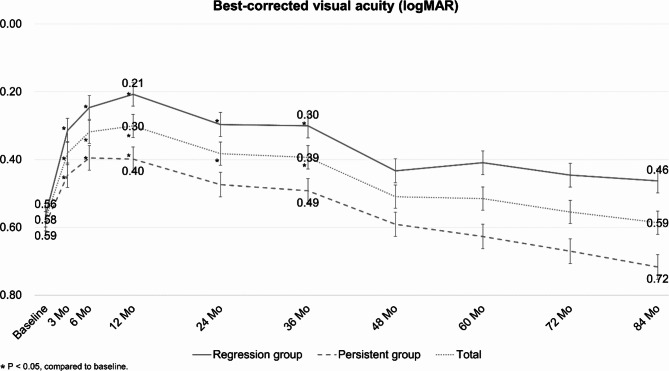
Changes in best-corrected visual acuity (BCVA) after the combination of photodynamic therapy with intravitreal anti-vascular endothelial growth factor (Anti-VEGF) injections for polypoidal choroidal vasculopathy

In all 33 eyes, the mean best-corrected visual acuity (BCVA) after combination treatment showed a significant improvement compared to the baseline BCVA up to 36 months (*p* = 0.003). However, no significant difference in BCVA compared to the baseline was observed at 48, 60, 72, and 84 months (*p* = 0.453, *p* = 0.448, *p* = 0.805, and *p* = 0.906, respectively). The visual acuity at 12 months was 0.30 logMAR, showing the highest level of improvement (Fig. 1).

In the regression group, the BCVA significantly improved from 0.56 ± 0.32 at the baseline to 0.30 ± 0.26 at 36 months (*p* = 0.001). The BCVA gradually decreased over time, and at 84 months, BCVA was 0.46 ± 0.50 with no significant difference compared to baseline. (*p* = 0.334). In the persistent group, the BCVA significantly improved from 0.59 ± 0.41 at the baseline to 0.40 ± 0.28 at 12 months (*p* = 0.006).Similarly, the BCVA gradually decreased to 0.72 ± 0.58 at 84 months (*p* = 0.551) (Fig. 1). The BCVA between the two groups differed significantly only at 12 months (*p* = 0.037). At 84 months, the BCVA of the regression group was superior to the persistent group, although there was no statistical significance between the two groups (*p* = 0.136).

**Fig. 2:**
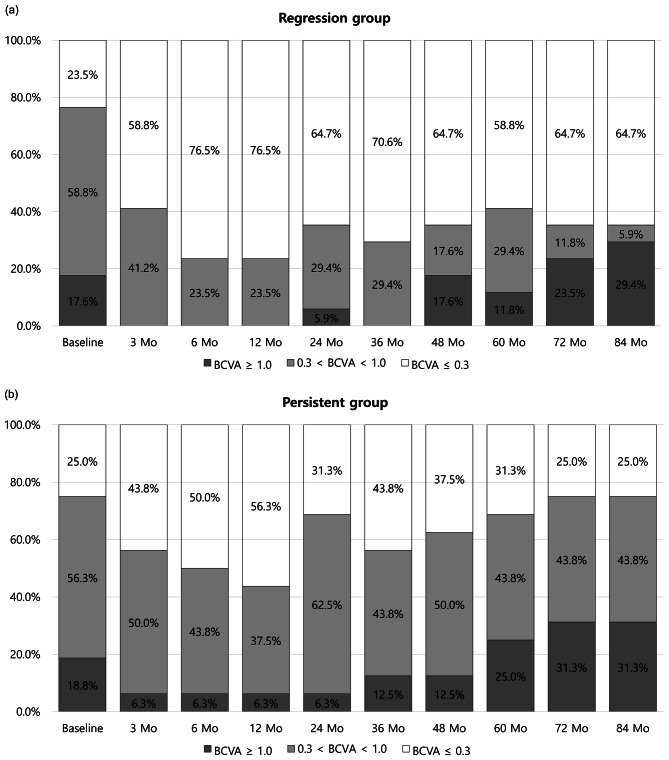
Distribution of best corrected visual acuity (BCVA) better than 0.3 logMAR and worse than 1.0 logMAR over 84 months. (**a**) In the regression group. (**b**) In the persistent group

At the baseline, there was no significant difference in the ratio of BCVA better than 0.3 logMAR between the regression group (23.5%) and the persistent group (25.0%) (*p* = 0.922). However, the difference gradually increased over time, reaching 64.7% in the regression group (Fig. 2a) and 25.0% in the persistent group (Fig. 2b) at 72 months (*p* = 0.022) and 84 months (*p* = 0.022).

### Changes in the CFT

Overall, the mean CFT showed the largest decrease at 6 months, from 342.6 ± 119.1 μm to 212.1 ± 51.7 μm. Although the mean CFT subsequently increased, it remained at a stable state throughout the follow-up period, significantly lower than the baseline level.

**Fig. 3:**
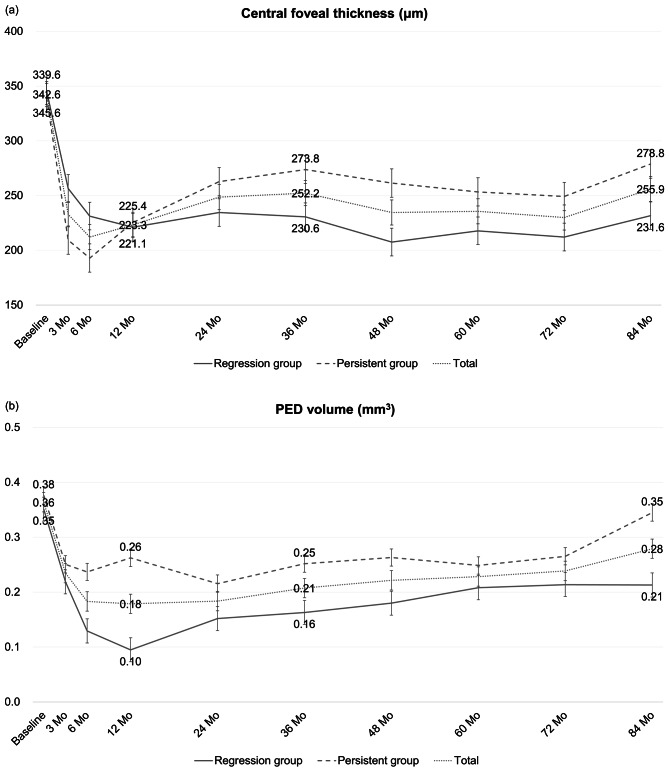
Changes in anatomical factors after the combination of photodynamic therapy (PDT) with intravitreal anti-vascular endothelial growth factor (Anti-VEGF) injections for polypoidal choroidal vasculopathy. (**a**) Changes in central foveal thickness (CFT). (**b**) Changes in pigment epithelial detachment (PED) volume

Subgroup analysis also revealed significantly decreased CFT compared to baseline during the follow-up period, except at 36 and 84 months in the persistent group (*p* = 0.079 and 0.074, respectively). During 84 months, the two groups exhibited no significant difference (Fig. 3a).

### Changes in the PED volume

The mean PED volume of total eyes showed the largest decrease to 0.18 ± 0.22 mm3 after 12 months of the combination treatment. The mean PED volume gradually increased over time, and at 84 months, there was no significant difference compared to the baseline (*p* = 0.289).

In regression group, the largest decrease in PED volume was observed at 12 months of 0.10 ± 0.14 mm3, and it remained relatively stable after 60 months. Although the PED volume was maintained after 60 months, there was no significant difference at 84 months compared to the baseline (*p* = 0.061). In persistent group, the largest decrease in PED volume was observed at 24 months, but the difference was not significant (*p* = 0.075). The PED volume gradually increased during the 84-month follow-up to 0.35 ± 0.33 mm3 (*p* = 0.589) (Fig. 3b).

### Retreatment

At 12 months, the PDT was administered 1.1 ± 0.3 times in regression group and 1.1 ± 0.3 times in persistent group (*p* = 0.790). The intravitreal anti-VEGF injection was administered 3.3 ± 0.8 times in regression group and 3.9 ± 1.4 times in persistent group (*p* = 0.179). During 84 months of follow-up, the PDT was administered 1.4 ± 1.0 times in the regression group and 1.9 ± 1.5 times in the persistent group (p = 0.444). The intravitreal anti-VEGF injection was administered 8.5 ± 7.1 in the regression group and 13.8 ± 15.2 in the persistent group (*p* = 0.326). At both 12 and 84 months, there was no significant difference in the number of PDT and anti-VEGF treatments between the two groups. The number of treatments administered each year is presented in Table 2.

**Table 2:** The number of treatments in the regression, and persistent groups

	Regression		Persistent	
	PDT	Anti-VEGF	PDT	Anti-VEGF
12 Mo	1.1 ± 0.3	3.3 ± 0.8	1.1 ± 0.3	3.9 ± 1.4
24 Mo	0.1 ± 0.3	1.2 ± 1.3	0.3 ± 0.6	1.9 ± 2.4
36 Mo	0.1 ± 0.3	1.2 ± 1.8	0.3 ± 0.6	1.8 ± 2.8
48 Mo	0.1 ± 0.2	1.1 ± 2.1	0.1 ± 0.3	1.7 ± 2.4
60 Mo	0.0 ± 0.0	0.8 ± 1.6	0.1 ± 0.3	1.6 ± 2.2
72 Mo	0.0 ± 0.0	0.6 ± 1.2	0.1 ± 0.3	1.3 ± 2.7
84 Mo	0.0 ± 0.0	0.2 ± 1.0	0.0 ± 0.0	1.7 ± 3.1
Total	1.4 ± 1.0	8.5 ± 7.1	1.9 ± 1.5	13.8 ± 15.2

The table presents the mean ± standard deviation (SD)

The number of patients who did not require additional treatment until 12, 24, 36, 48, 60, and 72 months was 13, 8, 8, 7, 6, 6 and 6 eyes in the regression group (17 eyes) and 9, 7, 7, 6, 5, 4, and 4 eyes in the persistent group (16 eyes).

### Adverse events

No patient showed systemic adverse events, such as cerebral vascular accidents or thromboembolic events, associated with intravitreal anti-VEGF injection. Among the ocular complications, submacular hemorrhage (SMH) was found in 6 out of 33 eyes (18.2%). (2 eyes in the regression group (11.8%) and 4 eyes in the persistent group (25.0%)). No other complications, such as cataract, increased intraocular pressure, retinal detachment, intraocular inflammation, or retinal vein occlusion, were observed.

## Discussion/Conclusion

Several studies on the treatment of PCV have evaluated polyp regression as an important anatomical outcome associated with visual prognosis. Previous studies have discussed the association between persistent polyps and increased risk of SMH, as well as recurrent vision loss and the need for additional treatment [18].

Combination therapy with anti-VEGF antibody and verteporfin PDT has shown superiority in achieving polyp regression. While anti-VEGF injection suppresses angiogenesis and reduces vascular leakage, PDT induces thrombosis and directly damages vascular endothelial cells, leading to selective occlusion of blood vessels in polypoidal lesions [19]. Due to the distinct mechanisms of action, a synergistic effect is expected from the combination treatment, and many studies have investigated the efficacy of PDT and intravitreal anti-VEGF injections in combination [10, 11, 20, 21].

Previous studies have investigated the long-term visual prognosis of combination treatment. Kang et al. [12] reported visual improvement in 44.1% of patients after 12 months of combination treatment and in 41.2% after 36 months during a 3-year follow-up, suggesting that the improved visual acuity could be maintained for 3 years. However, there was a decline in visual acuity observed in 2.9% of patients after 1 year and in 11.8% after 36 months, indicating a worsening trend. Similarly, in this study, the effects of combination treatment gradually decreased over time, with a decline in the level of visual improvement. During the 84 months follow-up, the mean BCVA of the combination treatment showed a significant improvement that continued for 36 months, followed by a decreasing trend. At 12 months, compared to the baseline, 36.4% of patients showed an improved BCVA after combination treatment, with 47.1% in the regression group and 25.0% in the persistent group showing improvement during follow-up. However, the percentage of patients with improved BCVA gradually decreased over time. At 84 months, only 27.3% of all patients, 35.3% in the regression group, and 18.8% in the persistent group showed visual improvement. Polyp regression is an important treatment goal. However, there is little research on long-term clinical outcomes regarding the association between polyp regression and visual prognosis. The direct relationship between polyp regression and visual outcomes in PCV remains controversial. Koh et al. [10] reported the 6-month clinical outcomes in the EVEREST study, where they observed visual improvement regardless of polyp regression. Other studies have reported favorable results in terms of polyp regression and visual improvement with combination therapy [14]. Lee et al. [16] reported that frequent anti-VEGF treatment can lead to polyp closure, but the role of polyp regression in preventing future recurrences remains uncertain. They also noted that achieving polyp regression through verteporfin PDT does not ensure long-term maintenance of visual gains.

Kikushima et al. [22] previously reported a 7-year clinical outcome of combined treatment with PDT and intravitreal injections. They categorized patients into the remission and the retreatment groups based on the presence of additional treatment over 5 years. The remission group had higher baseline BCVA and significantly better visual acuity at the 7-year follow-up. While the current study focused on earlier response to treatment based on polyp regression at the one-year using ICGA, the polyp regression group also had a significantly higher proportion of good visual acuity (20/40 or better). However, reactivation of polypoidal lesions requiring additional treatment might cause potential deterioration in the long-term mean visual acuity.

This study compares 7-year clinical outcomes according to polyp regression. In the regression group, longer-lasting visual acuity improvement up to 36 months was confirmed. Comparing the two groups according to polyp regression, there was no significant difference in BCVA, except at 12 months. Although the mean BCVA was better in the regression group compared to the persistent group, the difference was not statistically significant. However, the rate of maintaining good visual acuity at 72 and 84 months was higher in the regression group (64.7%) compared to the persistent group (25.0%) with a significant difference (*p* = 0.022). Additionally, comparing the number of treatments for each group maintaining visual acuity 0.3 logMAR or better, the regression group had a lower number of treatments compared to the persistent group, but the difference was not statistically significant.(*p* = 0.831 for anti-VEGF, *p* = 0.851 for PDT).

PED, especially sharp-peaked PED, is a hallmark of PCV and can be visualized on OCT images. Several previous studies have analyzed pigment epithelial detachment (PED) for volumetric analysis of polyps. Fenner et al. [23] found that baseline PED volume, lower choroidal vascularity index (CVI), and a more rapid reduction in PED volume are associated with reduced disease activity at month 12. Consistent with this context, our study also observed a significant reduction in the initial PED volume in the group exhibiting resolution of polyps. In addition, Vyas et al. [24] identified that the baseline PED volume, PED height, choroidal vascular hyperpermeability (CVH), and CVI were significant imaging biomarker for the disease activity at month 12. Our study also found that baseline PED volume was smaller, and the GLD appeared shorter in regression group, but no statistically significant difference was observed. Additional research is needed to explore the anatomical structure of polyps and their implications for prognosis.

The number of treatments received by the persistent polyp group was higher, but there were no significant differences (*p* = 0.444 for PDT, *p* = 0.326 for anti-VEGF). However, it is widely accepted that polyp regression can lead to a reduction in the occurrence of subretinal fluid, SMH, and other complications. Further comparisons involving a larger number of cases would be necessary.

The potential complications of PDT in patients with PCV include post-PDT hemorrhage, massive suprachoroidal hemorrhage, RPE tears, and micro-rips at the margin of the PED [25–27]. The reported risk of SMH in patients with PCV after PDT ranges from 3 to 30.8% [25, 27–30]. In this study, SMH was observed in 18.1% of the patients, with 2 eyes in the regression group and 4 eyes in the persistent group. No other complications were detected in this study. However, at 84 months, the BCVA of the 6 eyes with SMH was measured as 1.22 ± 0.52, which was worse than the BCVA of the 27 eyes without SMH, 0.45 ± 0.45 (*p* = 0.002). Thus, care should be taken during follow-up in cases of SMH, as it may lead to a deterioration in long-term visual acuity.

The limitations of this study include the small number of patients and retrospective nature, which may have affected the statistical analyses. However, the 84 months of follow-up monitoring enables the evaluation of long-term visual prognosis according to polyp regression. Another limitation is the lack of assessment of the choroidal status, which is important aspect in understanding the pathogenesis of PCV. Further studies should be conducted using a long-term, well-randomized design with a larger number of participants.

In conclusion, the combination of intravitreal anti-VEGF injection and verteporfin PDT is an effective treatment for PCV. Eyes with polypoidal regression at 1 year exhibited sustained visual acuity improvement up to 36 months, and even after 7 years, a higher percentage of patients were able to maintain better visual acuity. Although there was no statistically significant difference in the mean visual acuity between the groups, this study suggest that polyp regression is associated with favorable long-term visual outcomes, especially maintaining good visual acuity.

## Data Availability

The datasets used and/or analyzed during the current study are available from the corresponding author on reasonable request.
